# Impact of social media interventions and tools among informal caregivers of critically ill patients after patient admission to the intensive care unit: A scoping review

**DOI:** 10.1371/journal.pone.0238803

**Published:** 2020-09-11

**Authors:** Stephana J. Cherak, Brianna K. Rosgen, Mungunzul Amarbayan, Kara Plotnikoff, Krista Wollny, Henry T. Stelfox, Kirsten M. Fiest

**Affiliations:** 1 Department of Community Health Sciences, Cumming School of Medicine, University of Calgary, Calgary, AB, Canada; 2 Department of Critical Care Medicine, Alberta Health Services, Calgary, AB, Canada; 3 O’Brien Institute for Public Health, University of Calgary, Calgary, AB, Canada; 4 Hotchkiss Brain Institute, University of Calgary, Calgary, AB, Canada; 5 Faculty of Nursing, University of Calgary, Calgary, AB, Canada; 6 Department of Psychiatry, Cumming School of Medicine, University of Calgary, Calgary AB, Canada; Universita degli Studi della Campania Luigi Vanvitelli, ITALY

## Abstract

**Background:**

The use of social media in healthcare continues to evolve. The purpose of this scoping review was to summarize existing research on the impact of social media interventions and tools among informal caregivers of critically ill patients after patient admission to the intensive care unit (ICU).

**Methods:**

This review followed established scoping review methods, including an extensive a priori-defined search strategy implemented in the MEDLINE, EMBASE, PsycINFO, CINAHL, and the Cochrane CENTRAL Register of Controlled Trials databases to July 10, 2020. Primary research studies reporting on the use of social media by informal caregivers for critically ill patients were included.

**Results:**

We identified 400 unique citations and thirty-one studies met the inclusion criteria. Nine were interventional trials–four randomized controlled trials (RCTs)–and a majority (n = 14) were conducted (i.e., data collected) between 2013 to 2015. Communication platforms (e.g., Text Messaging, Web Camera) were the most commonly used social media tool (n = 17), followed by social networking sites (e.g., Facebook, Instagram) (n = 6), and content communities (e.g., YouTube, SlideShare) (n = 5). Nine studies’ primary objective was caregiver satisfaction, followed by self-care (n = 6), and health literacy (n = 5). Nearly every study reported an outcome on usage feasibility (e.g., user attitudes, preferences, demographics) (n = 30), and twenty-three studies reported an outcome related to patient and caregiver satisfaction. Among the studies that assessed statistical significance (n = 18), 12 reported statistically significant positive effects of social media use. Overall, 16 of the 31 studies reported positive conclusions (e.g., increased knowledge, satisfaction, involvement) regarding the use of social media among informal caregivers for critically ill patients.

**Conclusions:**

Social media has potential benefits for caregivers of the critically ill. More robust and clinically relevant studies are required to identify effective social media strategies used among caregivers for the critically ill.

## Introduction

Social media is defined as “websites and applications that enable users to create and share content or to participate in social networking” [1]. Social media tools are platforms and communities, such as Facebook or Skype, that facilitate quick communication and enable interaction among several users at any given time [2]. Social media participation in older age groups is steadily increasing [3], contributing to over 3.2 billion active users worldwide [4]. In considering the various user-generated content and social networking platforms, the role of social media conveys different meanings between users and non-users, age groups (e.g., millennials), and demographic populations. Since technological change is associated with linguistic and cultural changes, the role of social media is constantly in flux [5].

The use of social media in healthcare for increasing speed of communication, distributing accurate information, and promoting knowledge of support, treatments and self-care options is becoming more widespread [6, 7]. Patient- and family-centered healthcare, which acknowledges that patients and their informal caregivers are central figures in decision-making and delivery of care [8], recognizes that patients and caregivers exist within an online social structure and network of relationships [9]. Social media tools, such as real-time communication platforms, educational material, and self-management guides, are now more commonly incorporated in the decision-making process to aid caregivers with making informed decisions regarding their loved one’s care [10].

Critically ill patients are often unable to communicate their care preferences (e.g., due to mechanical ventilation, coma, etc.) including those that are in line with their individual values and goals [11]. In these situations, critically ill patients rely on their informal caregivers to learn about their diagnosis and treatment options, and to make important decisions on their behalf [12]–these situations can be stressful and distressing for an informal caregiver [13]. Family-centered interventions may improve caregiver’s comprehension, satisfaction, and long-term psychological outcomes during and after a family member’s critical illness [13, 14]. Social media tools as family-centered interventions might allow for personalization, presentation, and participation of informal caregivers in their loved one’s care, engaging them in the decision-making process and promoting better patient and informal caregiver outcomes [2, 15]. Despite their potential value, it is unclear whether social media tools can be meaningfully and systematically deployed in critical care medicine [16]. We therefore asked the question: What is the extent, range, and nature of research evidence on the impact of social media interventions and tools among informal caregivers of critically ill patients?

## Methods

This scoping review was conducted and reported as per the Arksey-O’Malley 5-stage scoping review method [17]. The approach for this review followed the Scoping Review Methods Manual by the Joanna Briggs Institute [18]. The Preferred Reporting Items for Systematic Reviews and Meta-analysis Protocols (PRISMA-P) guideline was used to develop the protocol [19] (S1 Table). We adhered to the PRISMA-ScR Extension for Scoping Reviews [20] to report findings.

### Populations, settings, and study designs

Inclusion criteria were as follows: (1) primary quantitative or qualitative research; (2) reporting on social media use with at least one informal caregiver as an end-user; (3) conducted with informal caregivers of critically ill patients of any age group; and (4) in any language or publication year. Studies were excluded if they were not primary research (e.g., reviews or editorials), did not report on caregiver use of social media, or were not conducted in a critical care population. For the purposes of this review, we defined: (1) a caregiver as any informal (i.e., non-clinical) person who regularly provides support to the patient and is in some way directly implicated in the patient’s care or directly affected by the patient’s health problem (e.g., family, friend); (2) social media as any form of electronic communication that allow users to share information and other content and create online communities; and (3) critically ill patients as any persons who are currently admitted to an intensive care unit (ICU) or had previously been admitted to an ICU. Studies were excluded if only abstracts were available.

### Data sources and searches

Comprehensive literature searches were conducted in MEDLINE, EMBASE, PsycINFO, CINAHL, and the Cochrane CENTRAL Register of Controlled Trials. The search strategies for each database were developed with a Medical Librarian (DLL) and were revised after reviewing preliminary search results. The search strategies combined synonyms and subject headings from three concepts: 1) caregivers; 2) critical care; and 3) social media. A search of the Cochrane Database of Systematic Reviews was undertaken to identify review articles related to the research question and their reference lists were screened to identify potential studies missed in the search. All databases were searched from inception to July 10, 2020. Reference lists of included papers were reviewed to identify potential studies missed in the search. No language or date limits were applied. The complete MEDLINE search strategy is shown in S2 Table.

### Study selection

After a subset of the team (SC, MA) achieved 100% agreement on a pilot-test of 50 random studies, all titles and abstracts were reviewed independently in duplicate by two reviewers (SC, MA). Any study selected by either reviewer at this stage progressed to the next stage. The full-text of all articles was reviewed independently in duplicate by two reviewers (SC, MA); articles selected by both reviewers at this stage were included in the final review. Disagreements were resolved by discussion or the involvement of a third reviewer (BR) when necessary. References were managed in Endnote X9 (Clarivate Analytics, Philadelphia, PA, USA).

### Data charting

Two reviewers (SC, KP) abstracted data independently and in duplicate for each included study using a data collection sheet developed and piloted by the review team. Discrepancies were resolved through discussion with a third reviewer (MA). Information on document characteristics (e.g., year of publication, geographic location), study characteristics (e.g., setting), caregiver group (e.g., spouses, parents, family caregivers), social media tool used (e.g., communication platform, content community, social networking site, blog or microblog), objectives and outcome measures of social media use, statistical significance, and authors’ conclusions were collected. Studies that examined social media as one component of a complex intervention were noted as such.

### Data synthesis and analysis

Findings were synthesized descriptively to map different areas of the literature as outlined in the research question. Using a social media framework described in previous research [6], we categorized social media tools into five categories: collaborative projects (e.g., EndNote, Slack), blogs or microblogs (e.g., WordPress, Twitter), content communities (e.g., YouTube, SlideShare), social networking sites (e.g., Facebook, Instagram), and real-time communication platforms (e.g., Text Messaging, Web Camera, FaceTime) (S3 Table). Study objectives and outcomes were classified according to an adaptation from those outlined in Coulter and Ellins [21] proposed framework for strategies to inform, educate and involve patients (S4 Table). The main objective from each study was categorized into one of five categories: to improve health literacy, clinical decision making, self-care, patient safety or other. Outcomes reported in each study were classified as patient and caregiver knowledge, patient and caregiver experience, use of services and cost, health behaviors and health status, and usage feasibility. Studies that reported statistically significant outcomes determined by *p*<0.05 related to the main objective of the study were classified as “statistically significant.” Studies that reported outcomes that were not statistically significant were classified as “not statistically significant,” and if a study did not assess significance through statistical equations that study was classified as “not assessed.” Descriptive statistics were calculated using STATA IC 15 (StataCorp. *Stata Statistical Software*: *Release 15*. College Station, TX: StataCorp LLC).

## Results

**Fig 1:**
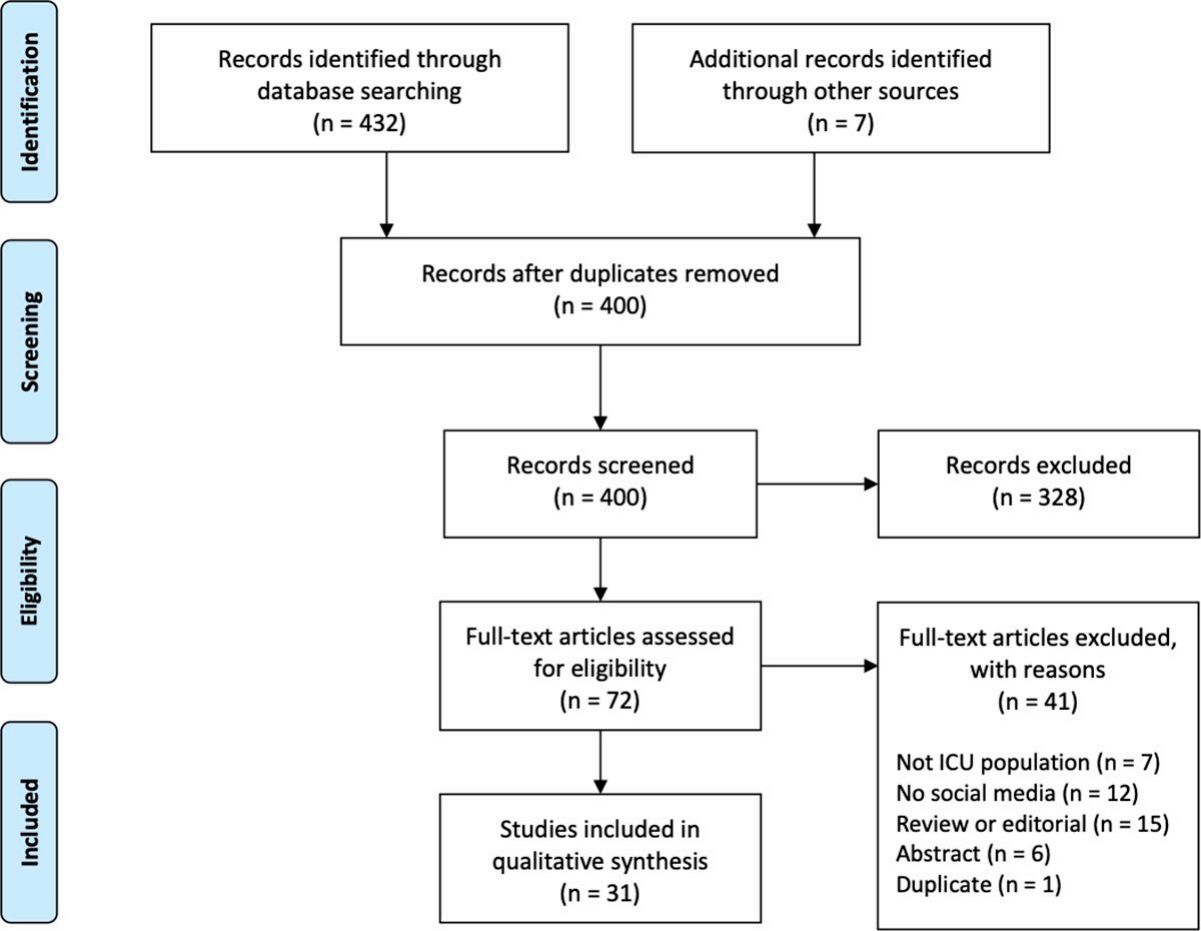
PRISMA diagram.

We screened 400 unique abstracts and reviewed 72 full-text articles; 41 full-text articles were excluded, the most common reasons being that the study did not report original research (n = 15/41) or that the study did not report on social media use (n = 12/41) (Fig 1). Hand searching resulted in the inclusion of seven additional studies. There was 85% agreement on title and abstract screening and 89% agreement on full-text screening.

### Description of included studies

**Fig 2:**
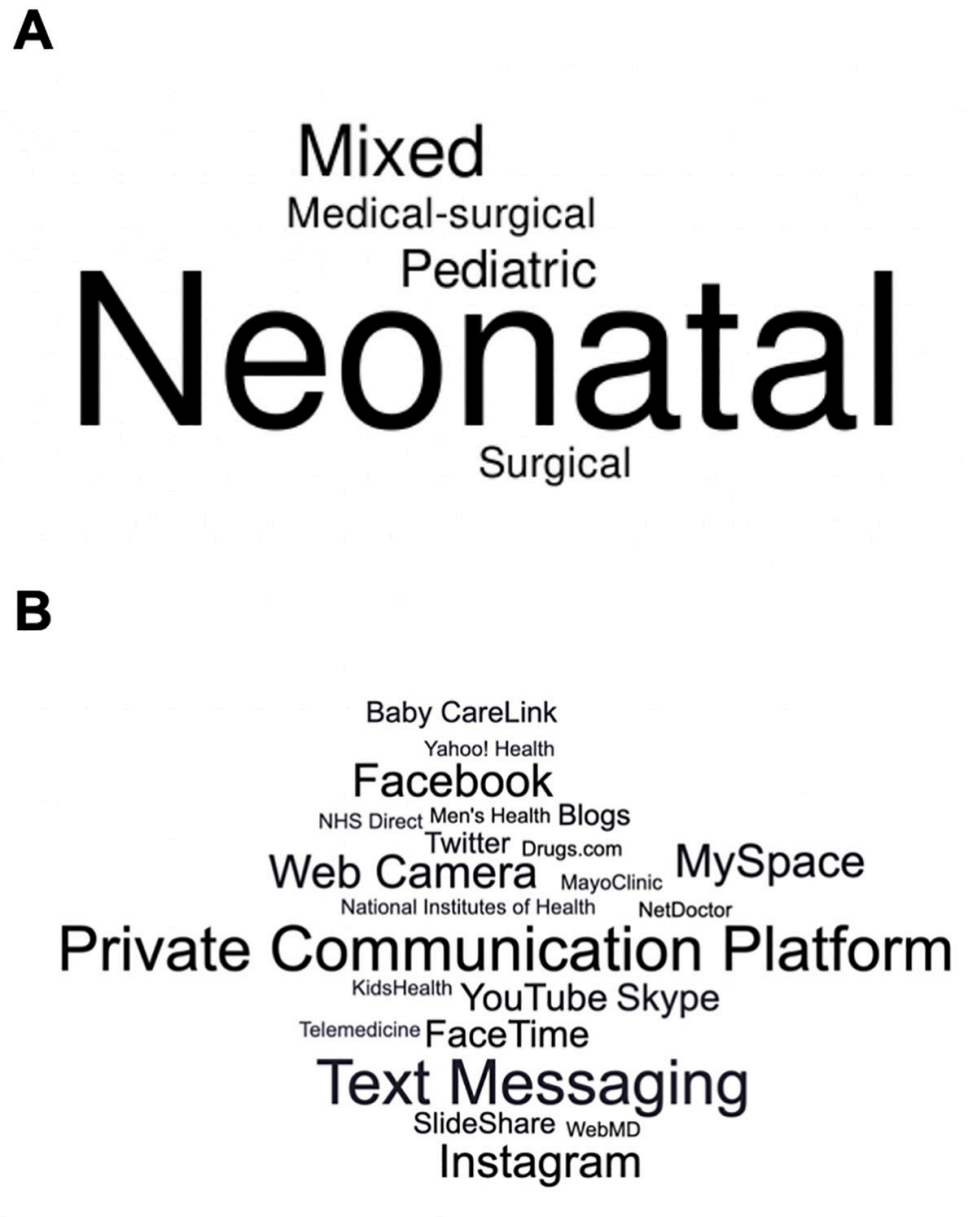
Representation of included studies. A ICU type; B specific social media tool in the included studies.

The 31 included studies [22–52] were published between 2000 and 2020 and primarily conducted in North America (n = 20, 65%) or Europe (n = 9, 29%), and with neonatal or pediatric critical care populations (n = 23, 74%) (Table 1). Fig 2A depicts the different ICU types from the included studies. The median start date was 2015 (range: 1997–2016) and the median duration was 19 months (range: 3–95 months). Many studies (n = 9, 33%) were interventional studies [22, 23, 27, 29, 30, 32, 33, 39, 48] of which most were conducted in neonatal ICUs (6/9). We included six qualitative studies and most (4/6) were conducted with neonatal or pediatric critical care populations. Caregivers were most commonly parents (n = 19, 61%) [30, 31, 33, 35–38, 41–44, 47–49, 51, 52] and unspecified family caregivers more broadly—which could include parents, but the term was more broadly defined (n = 7, 23%) [23, 24, 26, 27, 32, 39, 50]. One study was specific to mothers [40] and one study was specific to fathers [45]. Few studies reported additional perspectives from members of the clinical care team (e.g., nurses, primary care physicians) (n = 3, 10%) [29, 34, 50] or critical care patients (n = 3, 10%) [22, 28, 49]. More than half of the studies examined real-time communication platforms (e.g., FaceTime, Skype) (n = 17, 55%) [23, 24, 28, 30–40, 47, 51, 52], which accounted for many of the studies conducted with adult populations (3/7, 43%) and most of the studies conducted with neonatal or pediatric populations (14/22, 64%).

**Table 1:** Characteristics of included studies^1^.

**Adult Critical Care Populations**							
**Author, Year**	**Timeframe**	**N, Population**	**ICU Type**	**Design**	**Social media tool**	**Main study objective**	**Effects of social media, significance**
Das, 2019	2017–2018	473, Caregivers	Mixed	Cross-sectional survey	Social media in general	Clinical decision making	Positive, significant
de Havenon, 2015	2010–2012	88, Caregivers	Mixed	Non-randomized intervention	Communication platform	Caregiver satisfaction	Indeterminate, not significant
Hoffmann, 2018	2016	10, Caregivers; 10 Experts	Mixed	Qualitative content analysis	Communication platform	Health literacy	Positive, not assessed
Hetland, 2018	Not reported	374, Caregivers	Mixed	Qualitative content analysis	Social networking sites	Clinical decision making	Negative, not assessed
Loudet, 2017	2014	55, Patients; 39, Caregivers^2^	Medical-surgical	Prospective before-and-after	Content communities	Patient safety	Positive, significant
Mistraletti, 2016	2012–2013	332, Caregivers	Mixed	Prospective before-and-after	Social networking sites	Health literacy	Positive, significant
Nguyen, 2017	2013	169, Surrogate Decision Makers	Mixed	Prospective observation	Social media in general	Other^3^	Neutral, significant
Shiber 2016	Not reported	2, Patients; 2, Caregivers^2^	Surgical	Case study	Communication platform	Clinical decision making	Positive, not assessed
**Neonatal and Pediatric Critical Care Populations**							
**Author, Year**	**Timeframe**	**N, Population**	**ICU Type**	**Design**	**Social media tool**	**Main study objective**	**Effects of social media, significance**
Badke, 2019	2017	28, Parents	Pediatric	Cross-sectional survey	Content communities	Satisfaction	Positive, significant
Braner, 2004	2000–2003	73, Parents	Pediatric	Case-Series	Communication platform	Health literacy	Positive, significant
Coppola, 2013	Not reported	40, Parents	Neonatal	Prospective cohort	Social networking sites	Self-care	Neutral, significant
Epstein, 2015	Not reported	15, Parents	Neonatal	Prospective before-and-after, mixed methods	Communication platform	Caregiver satisfaction	Positive, significant
Flores-Fenlon, 2019	2013–2015	169, Parents	Neonatal	Cross-sectional survey	Communication platform	Caregiver satisfaction	Positive, not significant
Gabbert, 2013	2009–2010	141, Parents	Neonatal	Cross-sectional survey	Social networking sites	Other^6^	Neutral, not assessed
Globus, 2016	2012–2014	178, Parents; 62 Nurses	Neonatal	Prospective before-and-after	Communication platform	Caregiver satisfaction	Positive, significant
Gray, 2000	1997–1999	56, Parents	Neonatal	Randomized intervention	Content communities	Self-care	Positive, significant
Gund, 2013	Not reported	34, Caregivers	Neonatal	Randomized intervention	Communication platform	Caregiver satisfaction	Indeterminate, not assessed
Hughes Driscoll, 2020	2018–2019	59, Parents	Neonatal	Cross-sectional survey	Communication platform	Satisfaction	Indeterminate, significant
Jones, 2018	2005–2013	20, Parental Blogs	Pediatric	Qualitative thematic analysis	Blogs or microblogs	Self-care	Positive, not assessed
Joshi, 2016	2014	42, Nurses^2^	Neonatal	Prospective observation	Communication platform	Clinical decision making	Negative, significant
Kim, 2015	Not reported	25, Parents	Neonatal	Qualitative grounded theory	Communication platform	Other^4^	Neutral, not assessed
Kim, 2016	2014–2015	29, Paternal Blogs	Neonatal	Qualitative grounded theory	Social networking sites	Self-care	Neutral, not assessed
Lakshmanan, 2014	2009–2011	270, Parents	Neonatal	Cross-sectional survey	Content communities	Health literacy	Positive, significant
Lindberg, 2009	2006–2008	20, Parents	Neonatal	Qualitative thematic analysis	Communication platform	Caregiver satisfaction	Positive, not assessed
Orr, 2017	2013	72, Parents	Neonatal	Cross-sectional survey	Communication platform	Caregiver satisfaction	Positive, significant
Rhoads, 2015	2010–2012	320, Parents	Neonatal	Cross-sectional descriptive	Communication platform	Other^5^	Indeterminate, significant
Robertson, 2016	Not reported	12, Primary Care Physicians^2^	Neonatal	Randomized intervention	Blogs or microblogs	Health literacy	Negative, significant
Robinson, 2016	2012–2013	89, Caregivers	Neonatal	Randomized intervention	Communication platform	Self-care	Positive, significant
Safran, 2005	2003	235, Parents	Neonatal	Prospective cohort	Content communities	Patient safety	Positive, significant
Weems, 2016	Not reported	217, Mothers	Neonatal	Cross-sectional survey	Communication platform	Self-care	Indeterminate, not assessed
Williams, 2020	2018	41, Parents	Neonatal	Cross-sectional survey	Communication platform	Other^7^	Indeterminant, not assessed

^1^Categorized by ICU setting then sorted alphabetically first by first author last name.

^2^Reported at least one outcome related to social media use by an informal (i.e., non-clinical) caregiver; adult patient defined as >15 years.

^3^Reporting prevalence of internet use among critically ill septic patients and caregivers.

^4^Comparing mothers’ and fathers’ use of information and communication technology.

^5^Comparing mothers’ and fathers’ frequency and length of viewing their hospitalized neonate via webcam.

^6^Reporting prevalence of social networking site use among parents of preterm infants.

^7^Determining parents perception and preferences for information sharing in the neonatal intensive care unit.

### Social media tools

Included studies were categorized by the type of social media tool used (S3 Table). Fig 2B depicts the different specific social media tools from the included studies. Real-time communication platforms, that allowed user communication with messages, voice, and/or video, were the most common social media tool used (n = 15, 56%), followed by social networking sites (n = 6, 19%) and content communities (n = 5, 16%). Few studies (n = 2, 7%) assessed the use of blog or microblogs and only two studies examined social media use in general. Overall, most social media tools included functions that operated like communication platforms, such that they provided the option for users to post and share experiences. Many studies (n = 8, 30%) included a social media tool as part of a complex intervention, and most of these studies (n = 6/8) used mobile phones to facilitate the social media component. All of these studies (n = 6/6) reported that the ubiquitous nature and technical capacity of mobile phones were strong motivating factors. Several of these studies (n = 5/6) addressed potential misuse of information and privacy concerns over text messaging by an established mobile phone dedicated to the study, and provided recommendations to the clinical care team (i.e., nurses, physicians) for text messaging with informal caregivers.

### Objectives of social media use

The most common intended use of social media was for caregiver satisfaction (n = 9, 29%). Most studies that examined caregiver satisfaction used communication platforms (n = 8/9). Social networking sites were often used to improve self-care (n = 2/6, 30%), and content communities were mainly intended to improve patient safety (n = 2/4, 50%). There were few studies that addressed clinical decision making (n = 4, 13%) and half (n = 2/4) used content communities. Five studies (16%) did not fit the framework, and were classified as “other”; three of these studies reported the prevalence of social networking use (n = 1) or of internet use more broadly (n = 2), and two compared mothers and fathers use of information and communication technology (n = 1) or frequency and length of webcam viewing (n = 1).

### Outcomes and measures

**Fig 3:**
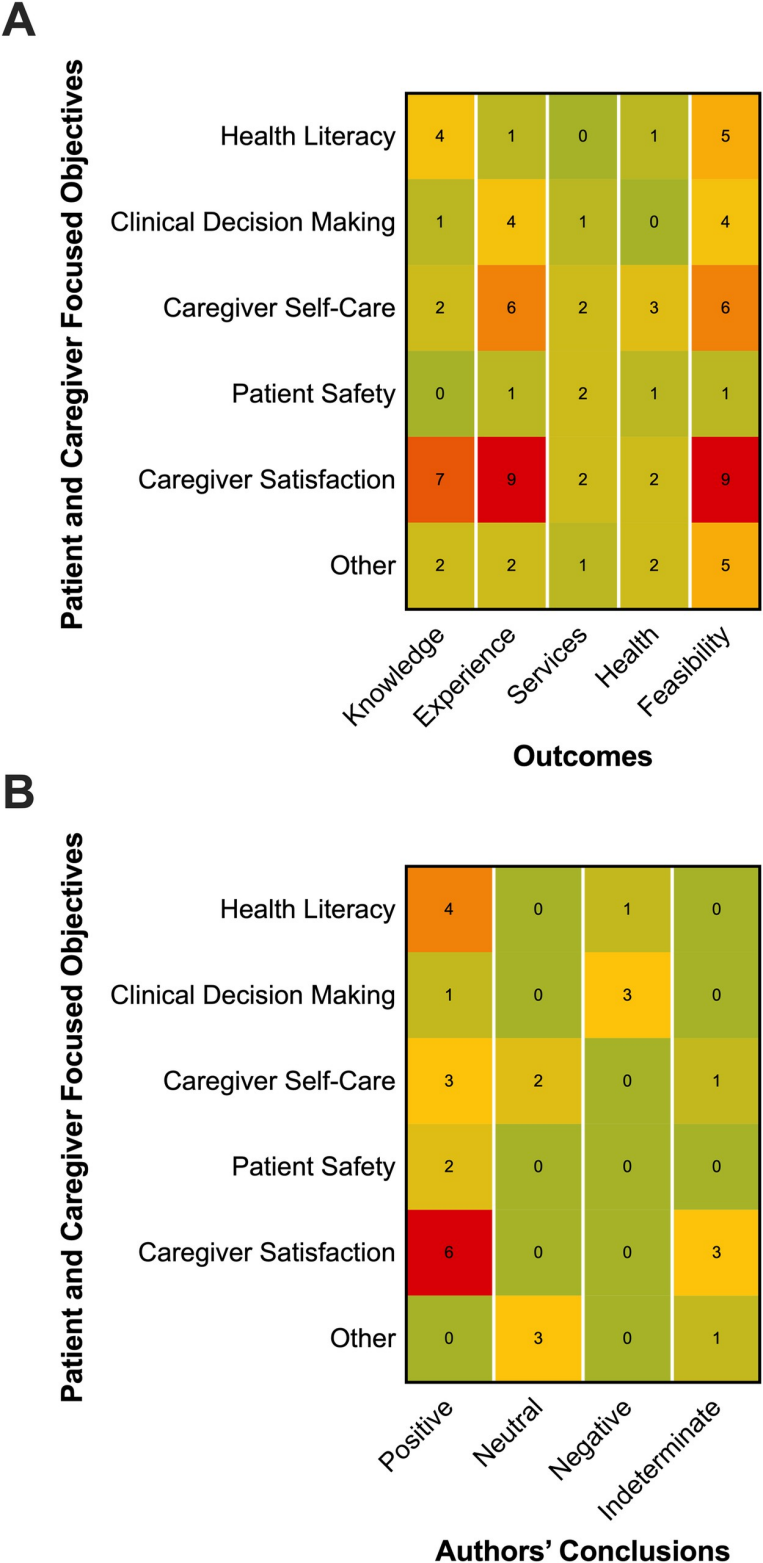
Summarized findings on social media outcomes. (A) patient and caregiver focused objectives^1,2,3^; (B) Authors’ conclusions on social media use with regard to patient and caregiver focused objectives^1,2,4^. ^1^Adapted from Coulter and Ellins, 2007; ^2^Only the main study objective was recorded from a single study; ^3^More than one outcome category could be recorded from a single study; ^4^Only one overall conclusion was recorded from each study. Frequency indicated by color: red, very frequent; yellow, moderately frequent; green, infrequent. N, number of studies.

Usage feasibility and patient and caregiver experience outcomes were most commonly reported (n = 30 and n = 23, respectively) (Table 2). Patient and caregiver knowledge outcomes were reported in 16 studies (52%), and use of services and cost outcomes, and health behaviors and health status outcomes were reported in eight studies each. Among outcomes related to usage feasibility (n = 30), measures of usage and demographics were most common (n = 22, 73%) and were often accompanied by measures of users’ attitudes and preferences (n = 20, 67%). Measures of patient or caregiver satisfaction or of clinician-patient/caregiver communication were most commonly reported for outcomes related to patient and caregiver experience (n = 13 and n = 12, respectively). Fig 3A provides a summary of outcomes as they relate to the study objectives. There were no defining trends between outcomes with regard to objectives for social media use, but measures related to the use of services and cost, or to health behaviors and health status, were generally least reported among any objective. One study reported outcomes related to potential for unintended consequences or harm from social media tools [50].

**Table 2:** Outcomes measured for social media use.

Outcomes^1^^,^^2^	N (%)^3^
Patient & Caregiver Knowledge	16 (52%)
Knowledge of condition and long-term complications	11 (35%)
Self-care knowledge	3 (10%)
Knowledge of treatment options and likely outcomes	9 (29%)
Comprehension of information	8 (26%)
Recall of information	3 (10%)
Patient & Caregiver Experience	23 (74%)
Patient or caregiver satisfaction	13 (42%)
Clinician-patient/caregiver communication	12 (39%)
Peer-to-peer communication	4 (13%)
Quality of life	2 (6%)
Psychological well-being	7 (23%)
Self-efficacy	3 (10%)
Involvement and empowerment	10 (32%)
Use of Services and Cost	9 (29%)
Hospital admission rates	4 (13%)
Emergency or ICU admission rates	4 (13%)
Length of hospital stay	4 (13%)
Number of visits to general practitioners	2 (6%)
Cost effectiveness	3 (10%)
Cost to patients or caregivers	1 (3%)
Days lost from work or school	0 (0%)
Health Behaviors and Health Status	12 (39%)
Health related lifestyles	1 (3%)
Self-care activities	1 (3%)
Treatment adherence	2 (6%)
Severity of disease and symptoms	4 (13%)
Physical functioning	2 (6%)
Mental functioning	4 (13%)
Clinical indicators	2 (6%)
Usage Feasibility	30 (96%)
Attitudes and preferences	23 (70%)
Content and accuracy	17 (54%)
Usability	18 (58%)
Usage and demographics	22 (71%)

^1^Adapted from Coulter and Ellins, 2007.

^2^More than one outcome measure was often reported in a single study.

### Evaluation of social media use

**Fig 4:**
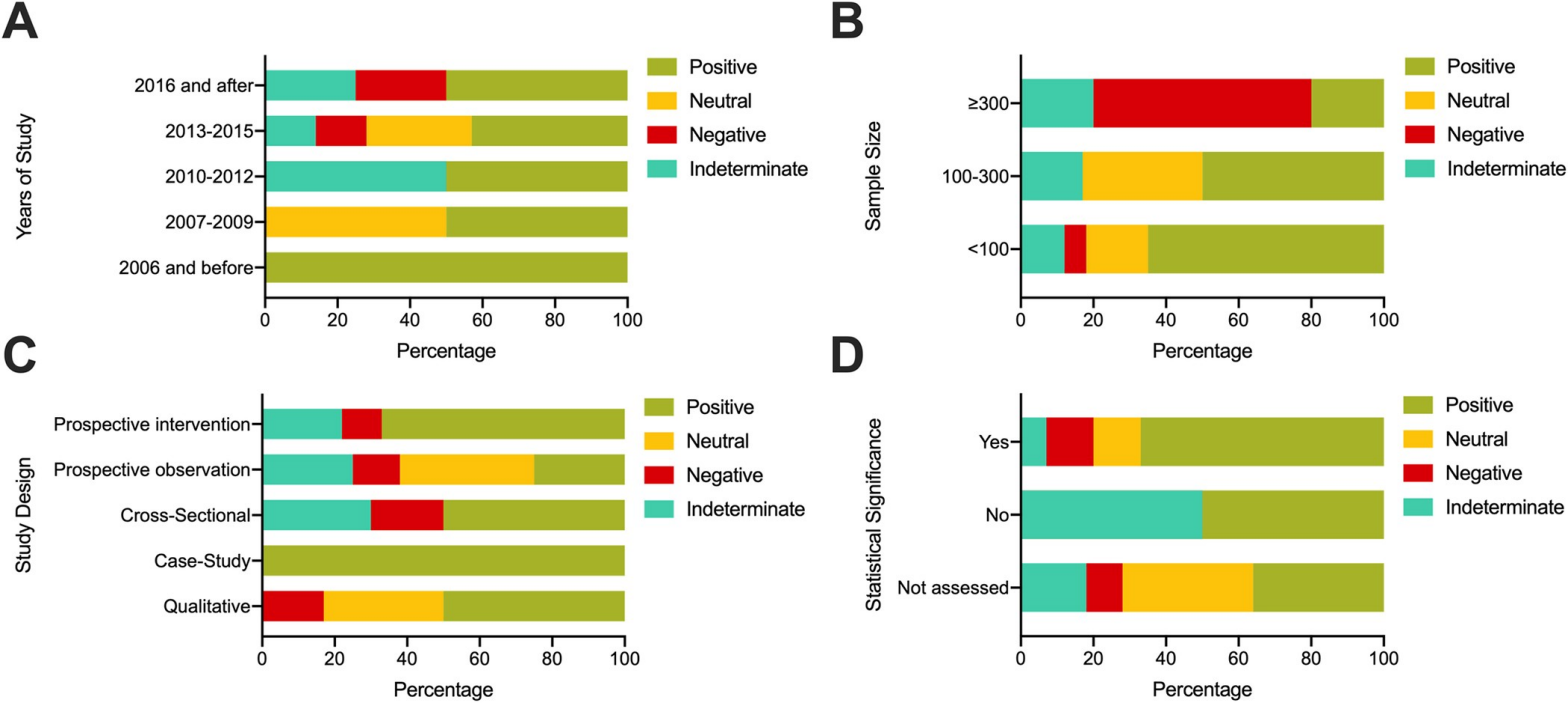
Authors’ conclusions. (A) years of data collection^1^; (B) sample size; (C) study design; (D) statistical significance. ^1^For eight studies year of publication was used as timeframe of data collection was not reported.

Fig 4 shows trends of authors’ conclusion by years of data collection, sample size, study design, and statistical significance. A positive effect of social media use was reported by majority of studies within each 2-year timeframe of years of data collection, except for 2013–2015 (Fig 4A). Studies that collected data during and/or after 2016 reported only positive, negative or indeterminate effects of social media use. Majority of studies with a sample size >300 reported a negative effect, and majority of studies with a sample size 100–300 or <100 reported a positive effect (Fig 4B). Prospective observational studies commonly reported a neutral effect and the majority of prospective intervention studies reported a positive effect (Fig 4C). Among the studies that assessed statistical significance, the majority determined that social media use had a positive effect (Fig 4D).

The most common type of study design was interventional (n = 9, 29%)—of which 4 were controlled by randomization (i.e., RCTs)—followed by prospective cohort (n = 8, 26%) and qualitative (n = 6, 19%). Of the quantitative studies (n = 25, 68%), majority assessed statistical significance (n = 20/25) and majority determined there was a significantly positive effect of social media use (n = 12/20). Among the randomized interventions (n = 4), two found a significantly positive effect, one found a significantly negative effect and one did not assess statistical significance. Fig 3B provides a summary of authors’ conclusions of social media use with regard to study objectives. The majority of studies with the objectives of improving health literacy, self-care, patient safety or caregiver satisfaction, reported a statistically significant positive effect. Among the four studies that aimed to improve clinical decision making, one study reported a positive effect but did not assess statistical significance, and three studies reported a negative effect but only two assessed significance.

## Discussion

We used scoping review methodology to synthesize the literature on the extent, range, and nature of research evidence on the impact of social media interventions and tools among informal caregivers of critically ill patients. There is a growing body of literature, primarily from neonatal or pediatric populations, suggesting that real-time communication platforms are now commonly used social media tools among informal caregivers of critically ill patients. In contrast, there is very little literature regarding caregiver use of social networking sites, blogs, or content communities. The most common intended use for social media was to improve caregiver satisfaction with the experience and role of an informal caregiver of a critically ill patient. Outcomes related to usage feasibility, such as measures of user’s attitudes, preferences, and demographics, were nearly always reported. Few studies assessed cost-effectiveness of using social media tools with informal caregivers, and outcomes related to health behaviors and health status of either the patient or caregiver were reported infrequently. Although most studies concluded that the use of social media among informal caregivers is beneficial and meaningful, the potential for unintended consequences or harm specific to informal caregivers were not adequately explored. The low reliability and high variability of content shared on social media highlights the importance of control from medical personnel to avoid the spread of “fake news” [53]. The emerging utilization of social media tools among informal caregivers for critically ill patients have practical implications for critical care medicine.

Modern mobile phones are powerful computational devices. The technical capacity of mobile phones to facilitate phone-based health interventions was a motivating factor for several included studies. Mobile phones are also omnipresent and nearly always at hand [54], which makes it possible to increase the number of points of care to virtually any place and time [55]. The combination of the technical capacity, personal nature, and convenient proximity of mobile phones has reduced barriers to adoption and increased acceptance of phone-based health interventions in numerous healthcare settings [56]. The immediacy of access of mobile phones might also be useful to informal caregivers after patient discharge by providing prompt advice and support, which may reduce healthcare costs by preventing hospital or ICU readmission.

Mobile phones in healthcare settings also have disadvantages. With regard to nursing, disruption of workflow, interruption of practice, and improper usage have been reported [57]. For example, in the study conducted by Piscotty and colleagues [58], 67% of nurses checked their mobile phone more than 2 times per shift and 22% checked their mobile phone more than 10 times per shift. Further, possibility of misuse of information that may violate patient privacy remains an unresolved problem [59]. Nursing organizations have responded with guidelines on professional social media use in the workplace [60–62]. Many included studies addressed potential privacy issues by an established mobile phone dedicated to the study, and recommended to refrain from using patient last names and conditions, to keep communications brief, and to destroy caregiver phone numbers after patient discharge [63]. That mobile phones may be useful to facilitate social media interventions in critical care medicine is a noteworthy finding of this review, but further research is needed on how social media strategies can be implemented into practice without violating privacy or ethical considerations.

Support and encouragement can contribute to caregiver confidence, which can promote better understanding of a stressful illness-related situation and enable the caregiver to provide better care [64]. Many included studies found that caregivers reported a more satisfactory critical care experience and increased knowledge of a patient’s condition and long-term treatment options when provided with links to online resources with credible information. In the last decade, several members of the United States Critical Care Societies Collaborative have started using social media [65]. The Society of Critical Care Medicine is one member, which uses web-based education initiatives to provide accurate and reliable information to educate their members and the public [15]. As well, The World Federation of Societies of Intensive and Critical Care Medicine also recognized that social media plays a large role in achieving more and better involvement with other member societies, and actively uses social media to liaise with important groups, such as young clinicians [66]. Considering the differences in how critical care societies use diverse approaches to deliver overlapping educational content can provide a rich opportunity to inform development of future web-based education initiatives, targeted specifically at informal caregivers.

Real-time communication platforms have been studied and implemented in many healthcare settings [67, 68]. Several included studies found that in neonatal ICU populations, parents who were communicating with the clinical care team using videoconferencing instruments (e.g., FaceTime, Skype) felt significantly more satisfied with their infants’ care when they were unable to be physically present. No study conducted in adult ICU populations used a social media tool dedicated entirely to videoconferencing, although most social media tools included functions which operated similar to communication platforms. Further, no included study from any ICU reported the use of communication platforms to engage non-local family members or young children who may benefit from remote communication with their loved one. Since many communication platforms are free to download on most electronic devices and allow for multiple users at once, an important area for future research is the use of communication platforms by entire support groups of both adult and non-adult critical care patients. This type of research is warranted to determine if positive outcomes of communication platforms depend on whether the caregivers’ relationship to the patient is parent-child (i.e., parent providing support to children) versus child-parent (i.e., children providing support to parents).

It is important to recognize that social media tools are exactly that—tools—rather than a substitute for personal interaction with healthcare providers. Recent studies in other healthcare settings have found that patients’ value in-person interaction with healthcare providers more than social media communication, and that healthcare providers are regarded as the most important source of information [69]. Knowledge on the values and preferences of the clinical care team, however, is lacking, and a common concern of many clinicians is that information shared on social media may not always be accurate. More understanding on physician preferences and social media accuracy is important as physicians often rely on patients’ informal caregivers to make decisions regarding the patient’s care, which frequently contributes to caregiver psychological morbidity [70]. Individualized social media interventions adapted to caregiver preferences may improve caregiver’s satisfaction and psychological morbidity [13]. More research on accurate, proper and potential use of social media in critical care medicine is required before implementation into daily practice.

Our review indicates there is untapped potential for social media interventions and tools to provide personalized support to informal caregivers of the critically ill. We recommend future inquiry on this topic examine mental health interventions using social media to determine the effect of social media mental health interventions on psychological outcomes of informal caregivers of the critically ill. This information is particularly relevant to challenges related to restricted visitation and social isolation associated with the COVID-19 pandemic [71]. The large numbers of patients experiencing critical illness and visiting restrictions enacted to prevent the spread of COVID-19 complicate participation of informal caregivers in patient care and recovery [72]. These factors are likely to make mental health consequences of critical illness on informal caregivers more prevalent and severe [73, 74]. Social media interventions and tools may be an effective mode of mental health support for informal caregivers of critically ill patients.

This scoping review has several strengths. We conducted an extensive literature search and screened reference lists of included studies in order to identify the full breadth of available literature on social media use in critical care populations. The search was executed in five bibliographic databases and was not restricted by language or dates. It was intentionally broad to ensure that social media use across all critical care populations were included. We followed rigorous methodology defined by adherence to recommended protocols and reporting criteria for scoping reviews. Further, the interdisciplinary team of a critical care physician, a critical care nurse, and a psychiatric epidemiologist, offered complementary expertise and knowledge. In spite of these strengths, there are limitations to note. We did not search the grey literature nor did we search social media itself, and could have missed studies, though our search strategy was comprehensive and full-text hand searching was completed. As well, the lack of a universal definition for social media, since social media is a relatively new concept that is continually transforming, added complexity to the process of study selection. However, our broad inclusion of study design allowed us to produce a comprehensive summary of the state of the literature on social media use by informal caregivers in critical care medicine. Ultimately, the relatively rapid evolution of social media means studies on usage will nearly exclusively reflect social media use of the past. Though such studies are valuable, it is important to note that the medium of social media is evolving faster than it is being studied.

## Conclusions

There is a growing evidence base to support the use of social media among informal caregivers of critically ill patients. There is untapped potential for social media tools to provide personalized support to informal caregivers. Social media tools might enable informal caregivers to gain the knowledge that they need in order to feel empowered, involved, and satisfied. Social media users should exercise caution on applications and networking sites so as not to compromise patient privacy. In sum, social media represents a flexible medium to deliver health information, and the individualized support that caregivers can obtain through using social media may promote an invaluable collaborative relationship when caring for critically ill patients.

## Supporting information

S1 Table Preferred Reporting Items for Systematic review and Meta-Analyses extension for Scoping Reviews (PRISMA-ScR). (DOCX)

S2 Table Medline search strategy. (DOCX)

S3 Table Categorization of social media tools. (DOCX)

S4 Table Patient and caregiver focused objectives and outcomes. (DOCX)

S5 Table Summarized findings on social media outcomes with regard to patient and caregiver focused objectives. (DOCX)

S6 Table Authors’ conclusions on social media use with regard to patient and caregiver focused objectives. (DOCX)
