# Supplementary material for: Impact of social media interventions and tools among informal caregivers of critically ill patients after patient admission to the intensive care unit: A scoping review
Source: PLoS One. 2020 Sep 11;15(9):e0238803. doi: 10.1371/journal.pone.0238803 (PMC7485758; doi:10.1371/journal.pone.0238803)
Supplement: S2 Table — (DOCX) [file pone.0238803.s002.docx]

**S2 Table. Medline search strategy**

| **Population** | **Intervention** | **Outcomes** | **Combination and Limiters^1^** |
| --- | --- | --- | --- |
| Caregivers | Critical Care/ICU | Social Media |  |
| 1. Exp Caregivers/ 2. Exp Family/ 3. Family.mp 4. Families.mp. 5. Caregiv*.mp. 6. “care giv*”.mp. 7. Carer*.mp. 8. Spouse.mp. 9. “next of kin".mp. 10. Support person.mp. 11. “loved one*”.mp. 12. “Significant other*”.mp. 13. Partner.mp. 14. Relative.mp. 15. Proxy.mp. 16. Surrogate.mp. 17. Friend.mp. 18. Or/1-17 | 1. Exp Intensive Care Unit/ 2. “intensive care”.mp. 3. ICU.mp. 4. Critical* ill.mp. 5. “critical care”.mp. 6. Or/19-23 | 1. exp Internet/ 2. Internet.mp 3. 25 or 26 4. Electronic Mail/ 5. Electronic mail.mp 6. 28 or 29 7. Mass Media/ 8. mass media.mp 9. 31 or 31 10. Online Systems/ 11. online systems.mp 12. 34 or 35 13. Medical Informatics/ 14. medical informatics.mp 15. 37 or 38 16. Computers/ 17. computers.mp 18. 40 or 41 19. Search Engine/ 20. search engines.mp 21. 43 or 44 22. Computer Communication Networks/ 23. Computer Communication Networks.mp 24. 46 or 47 25. Information Dissemination/ 26. Information Dissemination.mp 27. 40 or 50 28. Therapy, Computer-Assisted/ 29. computer assisted therapy 30. 52 or 53 31. "Marketing of Health Services"/ 32. “marketing of health services”.mp 33. 55 or 56 34. Social Marketing/ 35. Exp Social Environment. 36. social market* or social environment.mp 37. 58 or 59 or 60 38. 30 or 33 or 36 or 39 or 42 or 48 or 51 or 54 or 57 or 61 39. 27 and 62 40. (website* or web site* or webpage* or web page*).mp 41. Googl*.mp. 42. Facebook*.mp. 43. Instagram.mp 44. Snapchat.mp 45. elluminate.mp 46. flickr.mp 47. moodle.mp 48. picsearch.mp 49. skype.mp 50. elluminate.mp 51. ustream.mp 52. Netflix.mp 53. reddit.mp 54. tumblr.mp 55. slack.mp 56. messenger.mp 57. “chat room”.mp 58. YouTube.mp 59. WebMD.mp 60. ((e or electronic) adj3 newsletter*).mp. 61. ("Web 2.0" or "Web 2").mp. 62. "Health 2.0".mp. 63. "Medicine 2.0".mp. 64. (Social adj3 network*).mp. 65. linkedin.mp. 66. blog*.mp 67. wiki*.mp 68. podcast*.mp 69. (rss adj3 (reader* or feed*)).mp. 70. (forum* adj3 (internet or web* or chat*)).mp. 71. user generated content.mp. 72. microblog*.mp 73. (twitter or tweet*).mp. 74. (("peer to peer" adj5 network*) or P2P).mp. 75. (social adj3 media*).mp. 76. i-phone*.mp. 77. myspace.mp. 78. smartphone*.mp. 79. Or/63,64-102 | 1. 18 and 24 and 103 2. Exp animals/ not humans.sh 3. 104 not 105 4. Limit 83 to (abstracts and “review articles”) 5. 106 not 107 |

^1^Population, intervention, and outcome terms were combined within-group with the Boolean operator OR and then population terms yield was combined between-group with the intervention, and outcome terms yields using the Boolean operator AND.
