# Supplementary material for: Impact of social media interventions and tools among informal caregivers of critically ill patients after patient admission to the intensive care unit: A scoping review
Source: PLoS One. 2020 Sep 11;15(9):e0238803. doi: 10.1371/journal.pone.0238803 (PMC7485758; doi:10.1371/journal.pone.0238803)
Supplement: S3 Table — (DOCX) [file pone.0238803.s003.docx]

**S3 Table. Categorization of social media tools**

| **Tool^1^** | **Description** | **Examples** |
| --- | --- | --- |
| Collaborative projects | Enables joint and simultaneous creation and collaboration on content by many end-users. | EndNote  Monday.com  Slack  Wikipedia |
| Blogs or microblogs | Websites with date-stamped entries typically managed by a single person providing opportunities for interaction. | TinyBuddha.com (blog)  WordPress (blog)  Tumblr (microblog)  Twitter (microblog) |
| Content communities | Allows for sharing of media content between users, including text, photos, videos, and presentations. | Flickr  Instagram  YouTube  SlideShare |
| Social networking sites | Enables user connection across personal information profiles. | Facebook  LinkedIn  MySpace  ResearchGate |
| Communication platforms | Allows real-time user communication with voice and/or video. | FaceTime  Skype  Videoconferencing |

^1^Adapted from Kaplan and Haenlein, 2010
