# Supplementary material for: Impact of social media interventions and tools among informal caregivers of critically ill patients after patient admission to the intensive care unit: A scoping review
Source: PLoS One. 2020 Sep 11;15(9):e0238803. doi: 10.1371/journal.pone.0238803 (PMC7485758; doi:10.1371/journal.pone.0238803)
Supplement: S4 Table — (DOCX) [file pone.0238803.s004.docx]

**S4 Table. Patient and caregiver focused objectives and outcomes**

| **Objective^1^** | **Strategies** |
| --- | --- |
| To improve health literacy | Provisions of printed leaflets and health information packages |
|  | Provision of computer based and internet health information |
|  | Targeted approaches for low health literacy in disadvantaged groups |
|  | Targeted mass media campaigns |
| To improve clinical decision making | Patient decision aids |
|  | Training for clinicians in communication skills |
|  | Coaching and question prompts for patients |
| To improve self-care | Self-management educations |
|  | Self-monitoring and self-administered treatment |
|  | Self-help groups and peer support |
|  | Patient access to personal medical information |
|  | Patient-centered telecare |
| To improve patient safety | Information to help choose safe providers |
|  | Patient involvement in infection control |
|  | Encouraging adherence to treatment regimens |
|  | Checking records and care processes |
| To improve caregiver satisfaction | Any strategy with the primary objective to address caregiver satisfaction |
| **Outcome^1^** | **Measures** |
| Patient & caregiver knowledge | Knowledge of condition and long-term complications |
|  | Self-care knowledge |
|  | Knowledge of treatment options and likely outcomes |
|  | Comprehension of information |
|  | Recall of information |
| Patient & caregiver experience | Patient or caregiver satisfaction |
|  | Clinician-patient/caregiver communication |
|  | Peer-to-peer communication |
|  | Quality of life |
|  | Psychological well-being |
|  | Self-efficacy |
|  | Involvement and empowerment |
| Use of services and cost | Hospital admission rates |
|  | Emergency or ICU admission rates |
|  | Length of hospital stay |
|  | Number of visits to general practitioners |
|  | Cost effectiveness |
|  | Cost to patients or caregivers |
|  | Days lost from work or school |
| Health behaviors and health status | Health related lifestyles |
|  | Self-care activities |
|  | Treatment adherence |
|  | Severity of disease and symptoms |
|  | Physical functioning |
|  | Mental functioning |
|  | Clinical indicators |
| Usage feasibility | Attitudes and preferences |
|  | Content and accuracy |
|  | Usability |
|  | Usage and demographics |

^1^Adapted from Coulter et al., 2004
