# Supplementary material for: Impact of social media interventions and tools among informal caregivers of critically ill patients after patient admission to the intensive care unit: A scoping review
Source: PLoS One. 2020 Sep 11;15(9):e0238803. doi: 10.1371/journal.pone.0238803 (PMC7485758; doi:10.1371/journal.pone.0238803)
Supplement: S5 Table — (DOCX) [file pone.0238803.s005.docx]

**S5. Summarized findings on social media outcomes with regard to patient and caregiver focused objectives**

| **Objectives^1,2^** | | **Outcomes^1,3^** | | | | |
| --- | --- | --- | --- | --- | --- | --- |
|  |  | Patient & Caregiver Knowledge | Patient & Caregiver Experience | Use of Services and Cost | Health Behaviors and Health Status | Usage Feasibility |
|  |  | N=16 (52%)^4^ | N=23 (74%)^4^ | N=8 (26%)^4^ | N=8 (29%)^4^ | N=30 (97%)^4^ |
| Health Literacy | N=5 (16%)^4^ | 4 | 1 | 0 | 1 | 5 |
| Clinical Decision Making | N=4 (13%)^4^ | 1 | 4 | 1 | 0 | 4 |
| Self-Care | N=6 (19%)^4^ | 2 | 6 | 2 | 3 | 6 |
| Patient Safety | N=2 (6%)^4^ | 0 | 1 | 2 | 1 | 1 |
| Caregiver Satisfaction | N=9 (29%)^4^ | 7 | 9 | 2 | 2 | 9 |
| Other | N=5 (16%)^4^ | 2 | 2 | 1 | 2 | 5 |

^1^Adapted from Coulter and Ellins, 2007

^2^Only the main study objective was recorded from a single study

^3^More than one outcome category could be recorded from a single study

^4^N, number of studies that reported each objective or outcome category; %, N as a proportion of total included studies
