# Supplementary material for: Impact of social media interventions and tools among informal caregivers of critically ill patients after patient admission to the intensive care unit: A scoping review
Source: PLoS One. 2020 Sep 11;15(9):e0238803. doi: 10.1371/journal.pone.0238803 (PMC7485758; doi:10.1371/journal.pone.0238803)
Supplement: S6 Table — (DOCX) [file pone.0238803.s006.docx]

**S6. Authors’ conclusions on social media use with regard to patient and caregiver focused objectives**

| **Objectives^1,2^** | | **Conclusions^3^** | | | |
| --- | --- | --- | --- | --- | --- |
|  |  | Positive | Neutral | Negative | Indeterminate |
|  |  | N=16 (52%)^4^ | N =5 (16%)^4^ | N=4 (13%)^4^ | N=6 (19%)^4^ |
| Health Literacy | N=5 (16%)^4^ | 4  ↑ 3  ⇅ 0  ⇞ 1 | 0  ↑ 0  ⇅ 0  ⇞ 0 | 1  ↑ 1  ⇅ 0  ⇞ 0 | 0  ↑ 0  ⇅ 0  ⇞ 0 |
| Clinical Decision Making | N=4 (13%)^4^ | 1  ↑ 0  ⇅ 0  ⇞ 1 | 0  ↑ 0  ⇅ 0  ⇞ 0 | 3  ↑ 2  ⇅ 0  ⇞ 1 | 0  ↑ 0  ⇅ 0  ⇞ 0 |
| Self-Care | N=6 (19%)^4^ | 3  ↑ 2  ⇅ 0  ⇞ 1 | 2  ↑ 1  ⇅ 0  ⇞ 1 | 0  ↑ 0  ⇅ 0  ⇞ 0 | 1  ↑ 0  ⇅ 0  ⇞ 1 |
| Patient Safety | N=2 (6%)^4^ | 2  ↑ 2  ⇅ 0  ⇞ 0 | 0  ↑ 0  ⇅ 0  ⇞ 0 | 0  ↑ 0  ⇅ 0  ⇞ 0 | 0  ↑ 0  ⇅ 0  ⇞ 0 |
| Caregiver Satisfaction | N=9 (29%)^4^ | 6  ↑ 4  ⇅ 1  ⇞ 1 | 0  ↑ 0  ⇅ 0  ⇞ 0 | 0  ↑ 0  ⇅ 0  ⇞ 0 | 3  ↑ 1  ⇅ 1  ⇞ 1 |
| Other | N=4 (13%)^4^ | 0  ↑ 0  ⇅ 0  ⇞ 0 | 3  ↑ 1  ⇅ 0  ⇞ 2 | 0  ↑ 0  ⇅ 0  ⇞ 0 | 2  ↑ 1  ⇅ 0  ⇞ 1 |

^1^Adapted from Coulter and Ellins, 2007

^2^Only the main study objective was recorded from a single study

^3^Only one overall conclusion was recorded from each study

^4^N, number of studies that reported each objective or conclusion; %, N as a proportion of total included studies

↑, statistically significant; ⇅, not statistically significant; ⇞, statistical significance not assessed; Shading indicates majority statistically significant
